# Genome-Wide Investigation of *SBT* Family Genes in Pineapple and Functional Analysis of AcoSBT1.12 in Floral Transition

**DOI:** 10.3389/fgene.2021.730821

**Published:** 2021-09-07

**Authors:** Xingyue Jin, Yanhui Liu, Zhimin Hou, Yunfei Zhang, Yunying Fang, Youmei Huang, Hanyang Cai, Yuan Qin, Yan Cheng

**Affiliations:** State Key Laboratory of Ecological Pest Control for Fujian and Taiwan Crops, Fujian Provincial Key Laboratory of Haixia Applied Plant Systems Biology, College of Life Sciences, College of Plant Protection, College of Horticulture, Fujian Agriculture and Forestry University, Fuzhou, China

**Keywords:** subtilisin-like serine protease, pineapple, genome-wide, expression pattern, functional analysis

## Abstract

SBT (Subtilisin-like serine protease), a clan of serine proteolytic enzymes, plays a versatile role in plant growth and defense. Although *SBT* family genes have been obtained from studies of dicots such as Arabidopsis, little is known about the potential functions of SBT in the monocots. In this study, 54 pineapple *SBT* genes (*AcoSBT*s) were divided into six subfamilies and then identified to be experienced strong purifying selective pressure and distributed on 25 chromosomes unevenly. *Cis*-acting element analysis indicated that almost all *AcoSBT*s promoters contain light-responsive elements. Further, the expression pattern via RNA-seq data showed that different *AcoSBT*s were preferentially expressed in different above-ground tissues. Transient expression in tobacco showed that AcoSBT1.12 was located in the plasma membrane. Moreover, Transgenic Arabidopsis ectopically overexpressing *AcoSBT1.12* exhibited delayed flowering time. In addition, under the guidance of bioinformatic prediction, we found that AcoSBT1.12 could interact with AcoCWF19L, AcoPUF2, AcoCwfJL, Aco012905, and AcoSZF1 by yeast-two hybrid (Y2H). In summary, this study provided valuable information on pineapple *SBT* genes and illuminated the biological function of *AcoSBT1.12* in floral transition.

## Introduction

The normal function of plant cells is guaranteed by the precise regulation of protein level, which depends on the balance between protein synthesis and degradation (Schaller, 2004). Protein hydrolysis mediates protein degradation, not only enables amino acid recycling but also assists with post-translational modification (Have et al., 2018). Extensive proteolysis and site-specific limited proteolysis are the major mechanisms in protein hydrolysis. And the limited proteolysis, taking concerted action with other selective mechanisms, is essential for many plant biological processes such as subcellular trafficking, peptide hormone-regulated response, and immune response (Paulus et al., 2020).

Thus, evolutionarily, in order to ensure precise protein levels, a considerable number of limited proteases were widely distributed in plant cells. Among them, SBT (Subtilisin-like serine protease), a clan of serine proteolytic enzymes, were reported in numerous plants for their participation in the diverse cellular process such as protein activation. Structurally, SBTs own a highly conserved domain, named Peptidase_S8 domain (PF00082), which consists of a catalytic triad with the specific arrangement of three amino acid residues (Asp, His, and Ser) (Reichardt et al., 2018). This unique domain is served as the site of specific substrate binding (Schaller et al., 2018). Moreover, the protease-associated (PA) domain (PF02225) and the Inhibitor_I9 domain (PF05922) were also found in SBTs in plants. The conserved domains of SBTs are closely related to their versatile function evolved in plants.

Studies in model species have revealed that *SBT* genes exist throughout the plant kingdom and play versatile roles in plant growth and defense. *SBT* family has been well studied in Arabidopsis (*Arabidopsis thaliana*), and 56 SBTs were characterized. Among them, *AtSBT1.2* was found to be specifically expressed in stomatal precursor cells and function in stomata density regulation (Zhao et al., 2020). In *AtSBT2.4* deletion mutant ale1, the disrupted expression of *AtSBT2.4* leads to defects in leaf shape formation and embryo development (Creff et al., 2019). Moreover, it’s also reported that *AIR3* (*AtSBT5.3*) facilitates lateral root formation (Neuteboom et al., 1999), *ARA12* (*AtSBT1.7*) involves in seed coat development (Rautengarten et al., 2008), and *XSP1*(*AtSBT4.14*) regulates xylem differentiation (Zhao et al., 2000). Not only their functions in plant-specific developmental processes but also their involvement in Arabidopsis responses to environmental stress were reported. *AtSBT6.1*, encoding the protein necessary for bZIP17 activity in the endoplasmic reticulum stress signaling pathway, was observed to initiate salt stress response in plants (Liu et al., 2007). Additionally, loss-of-function of *AtSBT3.3* in deletion mutants compromises innate immune, while overexpression of *AtSBT3.3* enhances the plant pathogen-resistant (Ramírez et al., 2013). In soybean, *C1SBT* encoded protein exhibits stringent substrate specificity and induces the decomposition of soybean seed storage protein, whereas *SCS1* (*Seed Coat Subtilisin 1*) is preferentially expressed in soybean seed and involved in the remodeling of cell wall structure during seed coat development (Schaller et al., 2012). Also, in tomato, P69 identified as an SBT, induced by citrus exocytic viroid infection in tomato leaves, was found to behave as a pathogenesis-related protein which is important for plant-pathogen interactions (Schaller et al., 2018). More recently, the basal resistance protease tomato RCR3 is found to be activated by P69B (Paulus et al., 2020). Besides, 3 SBT genes (*SlPhyts3*, *SlPhyts4*, and *SlPhyts5*) in tomato were confirmed to trigger non-autolytic cell death under oxidative stress (Reichardt et al., 2018).

Pineapple (*Ananas comosus* L.) belongs to the family of Bromeliaceae, and it is one of the most important economic crops distributed across tropical and subtropical regions worldwide (Ming et al., 2015). In agriculture production, the flowering time of pineapples, which determines both the maximum reproductive success and the productivity of farmers’ labor, is very random and inconsistent (Bao et al., 2020; Jin et al., 2020). Although elucidation of mechanisms underlying pineapple flowering regulation will help maximize the food yield in the pineapple industry, few components regulating flowering time have been identified in pineapple. Our previous work adopted the systematic bioinformatics method WGCNA to analyze the spatio-temporal transcriptome data of pineapple floral organs and identified a series of pineapple *SBT* genes (*AcoSBT*s) located in flowering specific clusters (Wang et al., 2020). Moreover, this gene family is also reported in numerous cash crops for its versatile functions (Schaller et al., 2018; Paulus et al., 2020). Thus, it’ll be meaningful to carry out the fundamental study of the *SBT* gene family in pineapple. Recently, the assembled pineapple genome also provides an opportunity to genome-widely reveal the organization, evolution, and function of *AcoSBT*s (Ming et al., 2016).

In this study, a total of 54 *AcoSBT* genes were identified and classified into 6 subgroups based on their phylogenetic relationships. The results showed that pineapple *AcoSBT*s are located on 25 different chromosomes unevenly. We further investigated the exon-intron organization, motif structure, gene duplications, and expression profiles of AcoSBTs. Based on those bioinformatic analyses, we identified and functionally characterized the *AcoSBT1.12* in pineapple. Briefly, AcoSBT1.12 interacts with five candidate substrate proteins and negatively regulates floral transition. In summary, our study provided fundamental information on *AcoSBT*s and further determined the crucial role of *AcoSBT1.12* in controlling flowering time.

## Materials and Methods

### Identification and Characterization of SBTs in Pineapple

The Hidden Markov Model (HMM) of the Peptidase_S8 domain (PF00082) was obtained from the Pfam database^1^ as a query to search against pineapple genome database downloaded from Phytozome^2^ (Arango Argoty et al., 2012). After that, we further examined each of the selected candidates for having this conserved structure domain using NCBI-CDD^3^ (Marchler-Bauer et al., 2011). ExPAsy^4^ was then applied to calculate the molecular weights (MW) and isoelectric points (PI) of the AcoSBTs (Wilkins et al., 1999). Lastly, TargetP^5^ and SignalP^6^ were used for subcellular localization and signal peptide prediction, respectively (Emanuelsson et al., 2007).

### Sequence Alignment and Phylogenetic Analysis

All the identified pineapple SBT protein sequences were multiply aligned with Arabidopsis SBT sequences collected from TAIR^7^ using Clustal Omega with default parameters. Phylogenetic analysis was conducted by MEGAX with Neighbor-joining statistical method setting default parameter except for the bootstrap replications *n* = 1,000. A total of 54 AcoSBTs were classified into 6 different groups according to the AtSBTs’ group scheme. The evolutionary tree was visualized by the online tool iTOL^8^.

### Gene Structure, Motif Analysis, and *Cis*-Acting Elements Identification

The gene structure characteristics and exon-intron organizations of the AcoSBTs were exhibited using the TBtools program based on the comparison among the full-length genome sequences and the protein-coding sequences of the given genes (Chen et al., 2020). The web-based motif identification sever MEME (Mutiple Em for Motif Elicitation^9^) was used to detect potential motifs with following parameters: motif width < 50, motifs < 20, and *e*-value < e^−5^ (Granziol et al., 2019). Promoter regions, the 2,000 bp upstream regions of *AcoSBT* genes, were used for cis-element identification on web-based *Cis*-acting elements identification server PlantCARE^10^ (Lescot et al., 2002). The program Adobe Illustrator was adopted for result visualization.

### Chromosome Distribution and Evolutionary Analyses

The location information of all identified pineapple *SBT*s, including start positions, located chromosomes, and chromosome lengths, were collected from Phytozome for further visualization. BLASTP with the *E*-value < e^−5^ was used to search the potential anchors between pineapple and Arabidopsis, and the top 5 matches were identified. The syntenic blocks of *SBT*s from both pineapple and Arabidopsis were visualized by Circos (Krzywinski et al., 2009). Both *Ks* and *Ka* values were calculated with synonymous and non-synonymous substitutions options using Dnasp (Librado and Rozas, 2009).

### Plant Materials and Growth Conditions

The typical cultivated Pineapple (*Ananas comosus*) variety, MD2, was planted in plastic pots with long-day conditions: 30°C, 70% humidity, and 16 h light/8 h dark photocycle. MD2 pineapple organs (stamen, ovule, sepal, petal, and gynoecium) at different developmental stages were collected separately.

The Col-0 Arabidopsis was used to generate *AcoSBT1.12* transgenic lines. The seeds of wildtype and transgenetic lines were germinated on 1/2 Murashige and Skoog medium after surface-sterilized with 75% ethanol, followed by vernalization in a 4°C incubator for 48 h. Plates were then transferred in the greenhouse with controlled conditions as following: 28°C, 60% humidity, and 16 h light/8 h dark photoperiod. After 2 weeks, the healthy seedlings grown on the plates were selected and planted in plastic pots with soil under 16 h light/8 h dark photocycle for further experiments.

### RNA Extraction, RNA-Seq, and qRT-PCR

The developing samples of pineapple MD2 organs (sepal, petal, stamen, and ovule) were collected according to a previous paper (Wang et al., 2020). After sampling, three independent samples were collected and treated with liquid nitrogen rapidly, and then RNA extraction was performed immediately using the Trizol extraction kit (Omega Bio Tek, China) following the manufacturer’s guidance. The quality of RNA was confirmed by both agarose gel electrophoresis and spectrophotometer (NanoDrop, United States). The transcriptome data were downloaded from the NCBI database^11^ and the European Nucleotide Archive (accession number: PRJEB38680). The collected FPKM values (Fragments per kilobase of exon model per million mapped reads) were log_2_ transformed and visualized using the R package and genesis, respectively. qRT-PCR assays were carried out on the Bio-Rad Real-time PCR system (Foster, United States) using SYBR Premix Ex TaqII (Takara, Dalian, China), and the program was: 95°C for 30 s, 40 cycles of 95°C for 5 s, and 60°C for 30 s, followed by the last 95°C for 15 s. In each case, three technical replicates and three biological repeats were carried out. *AcoPP2A* (F: TTGTCATCGCTTCCTCCAAG; R: GTGTTGTCCACCACAGTATGA) and *AtACTIN* (F: TGCCAA TCTACGAGGGTTTC; R: TCTCTTACAATTTCCCGCTCTG) were selected as reference genes for pineapple samples and Arabidopsis samples, respectively.

### Vector Construction and Subcellular Localization

The *35S: AcoSBT1.12* vector was constructed as the following procedure: amplifying the full-length of *AcoSBT1.12* CDS sequence without termination codon and then cloning the amplified fragment into the Penter/D-TOPO vector, followed by recombining the positive clone into the destination vector pGWB605 harboring 35s promoter by LR reaction. The resulting vector was transformed into the *Agrobacterium tumefaciens* (GV3103) by electroporation and further transformed into Arabidopsis (Col-0) through the floral dip method. The multiple independent T1 plants confirmed by qRT-PCR were analyzed in further experiments. Above mentioned vector was introduced into *Nicotiana bethamiana* leaf epidermal cells by agroinfiltration. After 3 days growing at 28°C under 16 h light/8 h dark conditions, the leaves were subjected to the observation of the subcellular location of AcoSBT1.12-GFP under a Zeiss LSM750 confocal laser-scanning microscope.

### Yeast Two-Hybrid Assay

The coding sequence of *AcoSBT1.12* was cloned into pGBDT7 vector while the coding sequences of *AcoCWF19L*, *AcoPUF2, AcoCwfJL*, *Aco012905*, *AcoPM1P*, *Aco009239*, *AcoSZF1*, and *AcoCZF1* were cloned into pGADT7 vector, respectively. Then the AD and BD constructs were co-transformed into AH109 yeast strain. Firstly, the co-transformed yeasts were seeded on the synthetic dropout medium without Leu and Trp (SD/- Leu/- Trp), and then transferred to the synthetic dropout medium without Leu, Trp and adenine (SD/- Leu/- Trp/- His) for 4 days before observation.

### Statistical Analysis

Experiments were carried out with at least three biological repeats and three technical repeats, and Y2H was carried out with three technical repeats. The difference significances were statistically analyzed using the Student’s *t*-test. Asterisks denote significant differences between two groups of data (^∗^*P* < 0.05; ^∗∗^*P* < 0.01; ^∗∗∗^*P* < 0.001).

## Results

### Identification and Characterization of SBTs in Pineapple

To genome-widely identify SBTs in pineapple, the Hidden Markov Model (HMM) tool was used to search all the possible SBTs against the pineapple genome database with Peptidase_S8 domain (PF00082) as the query. After that, NCBI-CDD was employed to ensure that all the identified sequences contain the target domain (Marchler-Bauer et al., 2011). 54 AcoSBTs were identified in the pineapple genome and named according to their phylogenetic relationships with Arabidopsis SBT proteins. The length of 54 pineapple SBT proteins varied from 228 (AcoSBT5.9) to 2,759 aa (AcoSBT2.5), with the corresponding molecular weight ranged from 23.66 to 297.43 Kd. The isoelectric points (PI) of the AcoSBT varied from 5.29 (AcoSBT1.11) to 9.77 (AcoSBT1.15). Among them, 32 SBTs contain the plant-unique domain, PA domain, which is reported to determine the specificities of protein-protein interactions. In comparison, 40 SBTs possesses an additional Peptidase inhibitor I9 domain, which is characterized to function in the activation of the pro-enzyme, indicating the functional diversification of AcoSBTs (Arango Argoty et al., 2012). The detailed information about AcoSBTs, including transcript ID, protein size, chromosome location, and protein isoelectric point, were listed in Supplementary Table 1.

### Phylogenetic Analysis of Pineapple SBTs

**FIGURE 1:**
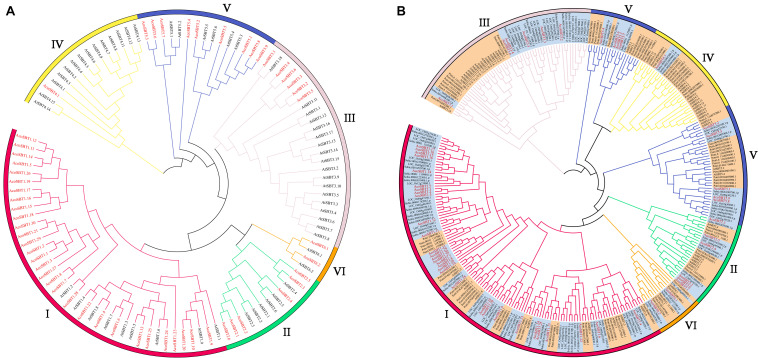
The evolutionary relationship of SBTs. **(A)** Phylogenetic relationship of the SBT proteins of Aco (Pineapple), At (*Arabidopsis thaliana*). **(B)** Phylogenetic relationship of the SBT proteins of Aco, At, Potri (*Populus trichocarpa*), Os (*Oryza sativa*), and Sbicolor (*Sorghum bicolor*). The phylogenetic tree was generated using neighbor-joining with 1,000. The phylo-genetic tree divides SBTs into six subfamilies, and the subfamilies are represented with different colors. Members from monocotyledonous plants are highlighted in orange while genes from dicotyledonous plants are highlighted in bright blue. SBT members from pineapple are marked in red font.

To investigate the evolutionary relationship of *AcoSBT*s, we constructed the phylogenetic tree of AcoSBT genes along with Arabidopsis SBTs using the Neighbor-joining statistical method. Full-length protein sequences of 56 Arabidopsis SBTs (AtSBTs) and 54 pineapple SBTs (AcoSBTs) were aligned to generate a neighbor-joining phylogenetic tree (Figure 1A). The SBT genes were divided into 6 subfamilies as previously reported, namely, Group I, Group II, Group III, Group IV, Group V, and Group VI. Among them, Group I was the largest subfamily with 9 AtSBTs and 30 AcoSBTs. Compared to Arabidopsis, the much greater number of SBTs in pineapple indicates that group I SBT members in pineapple might have a broader function and that Group I genes might have undergone the evolutionary divergence between dicotyledons and monocotyledons. The smallest group, Group VI, possesses only two genes in pineapple and Arabidopsis evenly, suggesting that this subfamily may not have undergone evolutionary divergence between dicotyledonous and monocotyledonous plants.

The evolutionary of SBTs between monocotyledons and dicotyledons were further explored based on the phylogenetic tree with monocotyledonous plants (*Ananas comosus*, *Oryza sativa*, and *Sorghum bicolor*) and dicotyledons (*Arabidopsis thaliana* and *Populus trichocarpa*) *SBT* genes (Figure 1B). In general, members from the same class tend to gather in the given subfamily. Among them, Group I is the largest subfamily, containing 126 members. Of the 126 members, 87 are monocots members accounting for 69%, and only 39 are dicots members accounting for 31%. Consistent with the speculation above, this disparity indicates Group I members probably experienced the differentiation between monocotyledons and dicotyledons. Moreover, Group VI is the smallest subfamily, with only seven genes in monocots and dicots evenly, further confirming the speculation that this subfamily is conserved in evolutionary divergence.

### Gene Structure Characterization and Protein Motif Identification

**FIGURE 2:**
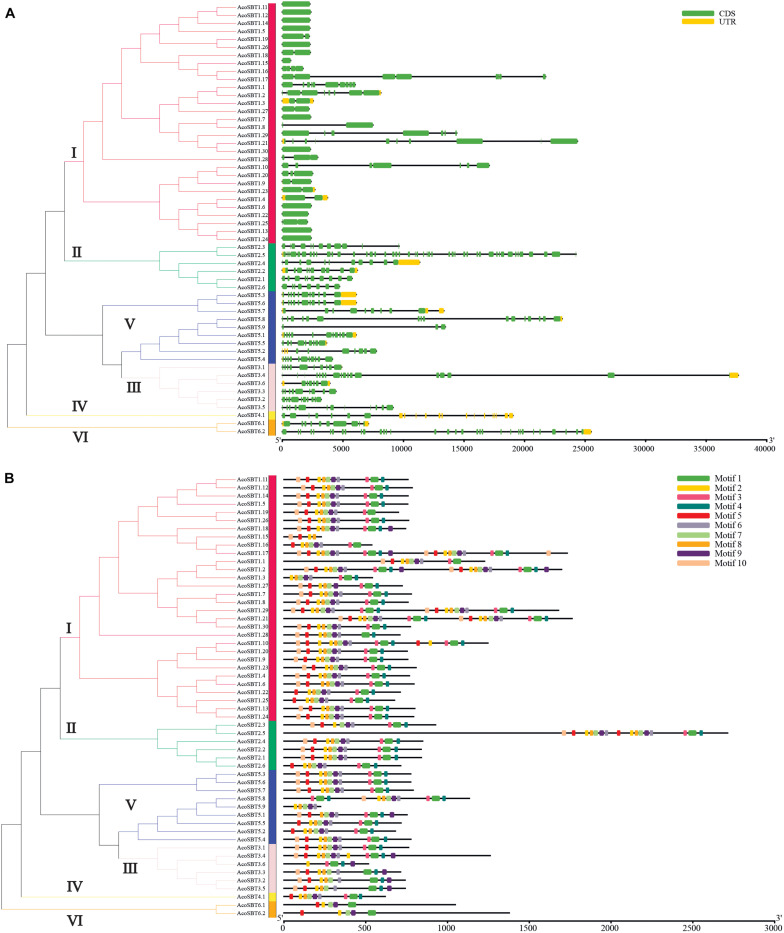
Gene structure and architecture of conserved protein motifs. **(A)** The Exon-intron structure of *AcoSBT*s. Green boxes represent CDS regions, yellow boxes represent UTR regions, and black lines represent untranslated introns. **(B)** The conserved motif composition of AcoSBTs. Different motifs are displayed by boxes with different colors.

To further understand their gene structure diversity, we analyzed the exon-intron organization of *AcoSBT* genes. As shown in Figure 2A, the exon numbers of the *AcoSBT*s range from 1 to 46. *AcoSBT2.5* contains the most exons, whereas 12 genes (*AcoSBT1.5*, *AcoSBT1.6*, *AcoSBT1.7*, *AcoSBT1.11*, *AcoSBT1.13*, *AcoSBT1.12*, *AcoSBT1.14*, *AcoSBT1.15*, *AcoSBT1.22*, *AcoSBT1.24*, *AcoSBT1.26*, and *AcoSBT1.30*) harbor only one exon. Genes from the same subgroup generally show similar gene structures, especially in the number and length of exons. All the genes with only one exon are from Group I, and most other Group I genes also contain fewer exons. All genes of Group II harbor more than 8 exons. This conserved gene structure of each subgroup suggests that *SBT* genes with high homologous sequence similarity tend to possess the same number of exons (Figure 2A).

Protein motifs are basic units of protein structure, which directly determined the function of given proteins. To elucidate the diversification of AcoSBT proteins, the conserved and diverged motifs were further identified by MEME with setting 10 motifs (Granziol et al., 2019). The detailed logo of each letter represents the level of conservation of amino acids (Supplementary Figure 1 and Supplementary Table 2). As displayed in Figure 2B, the number of motifs in pineapple SBT proteins ranged from 4 to 10. Motif 1 was founded in almost all AcoSBTs, except AcoSBT1.15, and AcoSBT5.9. For all AcoSBTs from Group II, their last motif on the C-terminal was motif 4, while almost all other group members ended with diverse motifs. 5 proteins (AcoSBT3.2, AcoSBT3.3, AcoSBT3.4, AcoSBT3.5, and AcoSBT3.6) from Group III, contained similar motif arrangement. Almost all the Group IV members, except AcoSBT3.1, ended with the conserved fragment of peptidases S8 domain Motif 9. The Group VI members showed the same protein structures in terms of motif number and motif type. For instance, members of this group did not preserve motif 10, which is acting as the “temporary inhibitors and assisting in the peptidase folding.” In conclusion, although a slight difference in motif arrangement was shown in some clusters, AcoSBTs from the same group generally possessed a similar protein structure.

### Chromosome Location and Evolutionary Analyses of *AcoSBTs*

All of 54 *AcoSBT* genes were distributed on 25 different chromosomes unevenly, as 7 genes were mapped onto linkage group (LG) 3, followed by 6 genes located in LG8, and 5 genes were distributed in LG7. While, 8 LGs, namely LG5, LG14, LG16, LG21, LG22, LG23, LG24, and LG25, were found to harbor only one *AcoSBT* gene.

**FIGURE 3:**
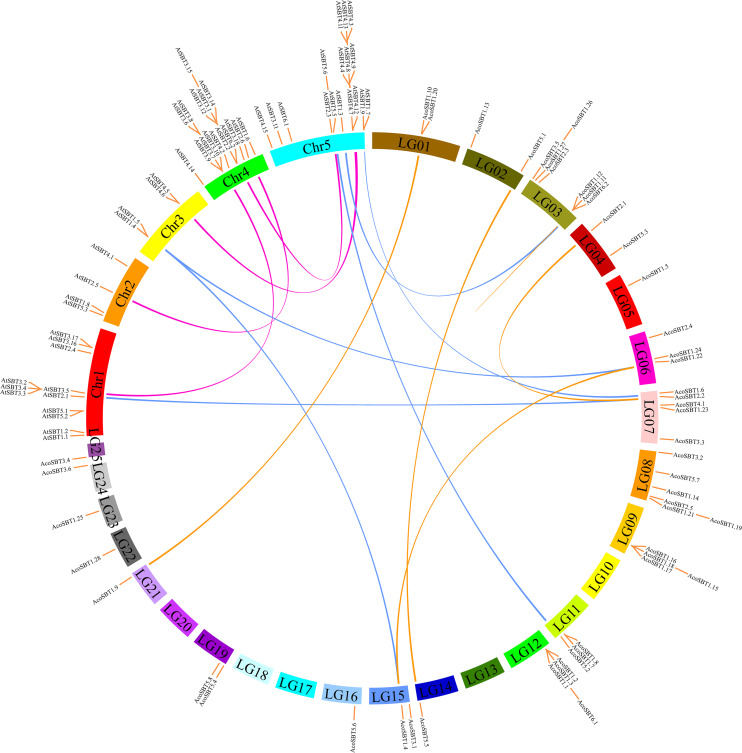
Synteny analysis of *SBT* genes between pineapple and Arabidopsis. Schematic representation for both chromosomal distribution and interchromosomal *relationships* of *SBT* genes.

To obtain the possible mechanism of the expansion of *AcoSBTs*, we investigated the gene duplication events in pineapple (Figure 3). A total of 5 duplicated pineapple SBT pairs indicating duplication events were identified: *AcoSBT1.10*/*AcoSBT1.9*, *AcoSBT5.1*/*AcoSBT5.5*, *AcoSBT2.1*/*AcoSBT2.2*, *AcoSBT1.22*/*Aco SBT1.4*, and *AcoSBT1.11*/*AcoSBT1.12*. Except for the gene pair *AcoSBT1.11*/*AcoSBT1.12*, which is a tandem duplication, all the other duplication events founded in the pineapple genome are segmental duplications. The result suggested that segmental duplication plays a more crucial role in the amplification process of *SBT*s in the pineapple genome. Comprehensive syntenic analysis of *SBT*s between pineapple and Arabidopsis was further conducted to illustrate the possible evolutionary mechanism of *AcoSBT*s. As a result, a total of 3 one-to-one syntenic orthologous gene pairs (*AtSBT2.1*/*AcoSBT2.2*, *AtSBT1.3*/*AcoSBT1.12*, and *AtSBT5.5*/*AcoSBT5.2*) were identified, suggesting that these genes derived from the same ancestor before the divergence of monocotyledonous plants and dicotyledonous plants. We also found other kinds of syntenic orthologous gene pairs, such as one *AcoSBT* corresponds to two *AtSBT*s (*AcoSBT1.6*-*AtSBT1.7*/*AtSBT1.9*), or one *AtSBT*s corresponds to two *AcoSBT*s (*AtSBT1.4*-*AcoSBT1.22*/*AtSBT1.4)*. These pairs might form after the divergence of monocotyledon and dicotyledon. We further calculated the *Ka/Ks* values of the gene pairs shown on the comparative synteny map (Supplementary Table 3). All the *Ka/Ks* values of *SBT* gene pairs are less than 1, with the highest value shown in the *AcoSBT1.22*/*AcoSBT1.4* pair (*Ka/Ks* = 0.506). These results suggested that the *SBT* gene family in pineapple experienced strong purifying selective pressure, as *Ka/Ks* > 1 means positive selection, *Ka/Ks* = 1 indicates neutral selection, and *Ka/Ks* < 1 represents negative selection (Zhang et al., 2020).

**FIGURE 4:**
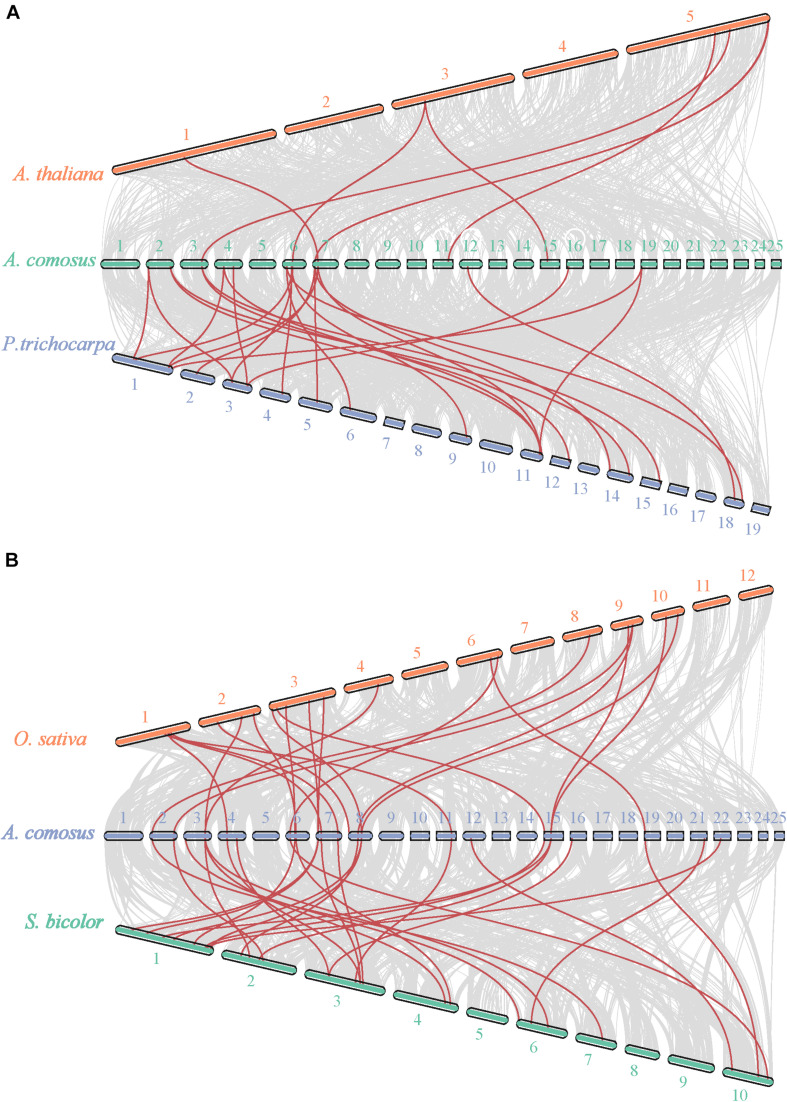
Synteny analysis of *SBT* genes between pineapple and Arabidopsis, populus, rice, and Sorghum. **(A)** Synteny analysis of *SBT* genes between pineapple and two dicotyledonous plants, Arabidopsis and populus. **(B)** Synteny analysis of *SBT* genes between pineapple and two monocotyledonous plants, rice and Sorghum. Gray lines in the background indicate the collinear blocks within pineapple and other plant genomes, while the red lines highlight the syntenic *SBT* gene pairs.

Given that comparative syntenic maps help to the study of evolutionary trait, we also have set up two comparative syntenic maps of pineapple associated with the above-mentioned four species, namely, Arabidopsis, Populus, rice, and Sorghum (Figure 4). According to the syntenic analyze results, we found 17 and 28 orthologous gene pairs in Arabidopsis and *P. trichocarapa*, while 28 gene pairs in both *O. sativa* and *S. bicolor* (Supplementary Table 3 and Figure 4). Some *AcoSBT*s had associated with multiple orthologous genes. Actually, many *AcoSBT*s from Group I are often corresponding to many *SBT*s from monocotyledons. For example, *AcoSBT1.11* is associated with *Sobic.002G161200*/*Sobic.006G176300* and LOC_Os04g47160, but we couldn’t find its orthologous gene in Arabidopsis and Populus. Additionally, *AcoSBT1.22* is associated with 3 monocots genes (*AcoSBT1.4*, *LOC_Os02g53860* and *Sobic.004G319000*) and 2 dicots genes (*Potri.001G167300* and *Potri.003G067000*), *AcoSBT1.4* is associated with 4 monocots genes (*LOC_Os09g26920*, *LOC_Os10g25450*, *Sobic.001G258100*, and *Sobic.002G216000*) but not with dicots gene. The results support our speculation in the evolutionary tree analysis that the Group I members may have undergone evolutionary divergence between dicotyledonous and monocotyledonous species. However, similar numbers of orthologous genes could not be found in Group VI. For example, *AcoSBT6.1* existed one gene pair in *P. trichocarapa* (*Potri.018G081400*) and *S. bicolor* (*Sobic.010G051200*), indicating these group genes may appear before the divergence of dicotyledonous and monocotyledonous. We calculated the *Ka*, *Ks*, and *Ka/Ks* of the gene pairs (Supplementary Table 3) and found that all the *Ka/Ks* values of *SBT* gene pairs are less than 1, further confirming that the *SBT* gene family experienced strong negative selective pressure.

### *Cis*-Acting Elements Identification in Promoter Region of AcoSBTs

**FIGURE 5:**
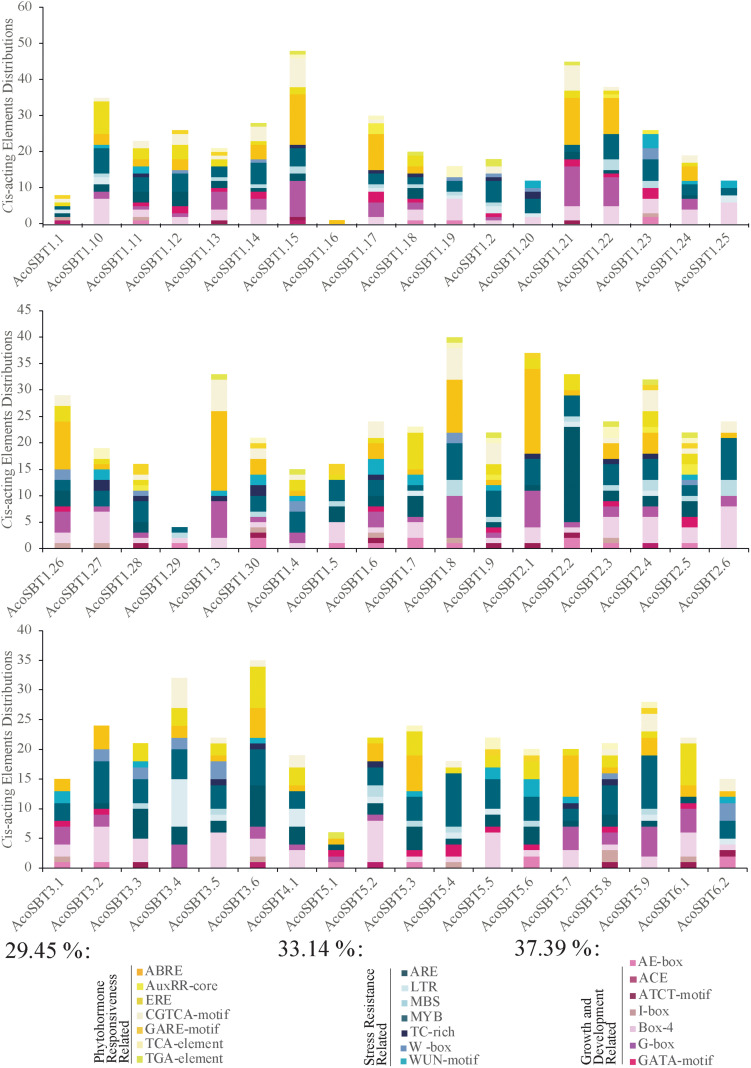
Information of *Cis*-acting elements in promoter region *AcoSBT* genes. Distribution, classification and proportion of *Cis*-acting elements in the promoter region of each gene.

To detect the potential biological functions of *AcoSBT* genes in pineapple, we collected the 2,000 bp upstream regions of all *SBT* genes and identified their *Cis*-acting elements using the PlantCARE program (Lescot et al., 2002). Within these 2,000 bp regions, 8 genes (*AcoSBT1.1*, *AcoSBT1.2*, *AcoSBT1.9*, *AcoSBT1.10*, *AcoSBT1.16*, *AcoSBT1.28*, *AcoSBT1.29*, and *AcoSBT5.1*) have overlapped regions with their upstream genes; thus, those overlapped regions were deleted, and the remaining promoter sequences were used for cis-elements analysis. A total 1,246 *Cis*-acting elements of 21 types were identified in the promoter region of 54 genes, classified into three categories: plant growth and development response elements, stress-related response elements, and phytohormone-related response elements (Figure 5, Supplementary Table 4, and Supplementary Figure 2). The plant growth and development response elements category include 7 types of Cis-acting elements (AE-box, ACE, ATCT-motif, I-box, Box-4, G-box, GATA-motif). And the top three *Cis*-acting components in this category are Box 4 (42.16%), G-box (32.39%), and GATA-motif (9.76%) (Figure 5 and Supplementary Table 4). Interestingly, all the three elements with the highest proportion belong to the parts of the module involved in light responsiveness (Jeong and Shih, 2003; Mande et al., 2018; Zheng et al., 2021). Moreover, only 20.92% of identified elements were classified into the stress-related response elements category. In this category, elements essential for the anaerobic induction (ARE, 37.7%), wound-responsive elements (WUN-motif, 15.58%), MYB binding site involving in drought-inducibility (MBS, 15.21%), elements involving in low-temperature reaction (LTR, 11.59%), elements responsive to pathogens (W-box, 11.23%), and elements related to defense responsiveness (TC-rich repeats, 8.70%) were identified. 32.20% of elements in the third category are phytohormone-related response elements category. Most of the elements in this category were responsive to abscisic acid (ABRE, 41.59%), ethylene (ERE, 23.00%), methyl jasmone (CGTCA-motif, 20.79%), auxin (TGA-element, 3.27%; AuxRR-core, 2.33%), and gibberellin (GARE-motif, 3.27%), etc.

To further investigate whether the *Cis*-acting elements were correlated with phylogenetic groups, we analyzed the *Cis*-acting elements for different subgroups. The results showed that the distribution of the *Cis*-acting elements in the promoter region did not show a strong correlation with phylogenetic groups. *AcoSBT1.15*, which possesses 48 *Cis*-acting elements, is the gene with the most *Cis*-acting, including 14 ABRE, 10 G-box, 8 CGTCA-motif. ABRE, CGTCA-motif, and TGAGC-motif are all components of phytohormone responsiveness, suggesting that *AcoSBT1.15* may have potential functions in the plant hormone pathway. With 45 *Cis*-acting elements, *AcoSBT1.21* harbored the second large number of *Cis*-acting elements, mainly composed of 13 ABRE, 11 G-box, and 7 CGTCA-motifs, indicating that A*coSBT1.21* may function in both the phytohormone responsiveness and light-responsive development. Moreover, as shown in Figure 5, Box 4, a conserved DNA module involved in light responsiveness was identified in almost all of the *AcoSBT* genes. Take together, it is suggested that the above study implies that the *AcoSBT* gene family may be closely associated with light-responsive growth and development in pineapple, which will provide clues for further studies on SBTs in pineapple.

### Spatio-Temporal Expression Profiles of *AcoSBT*s in Pineapple

**FIGURE 6:**
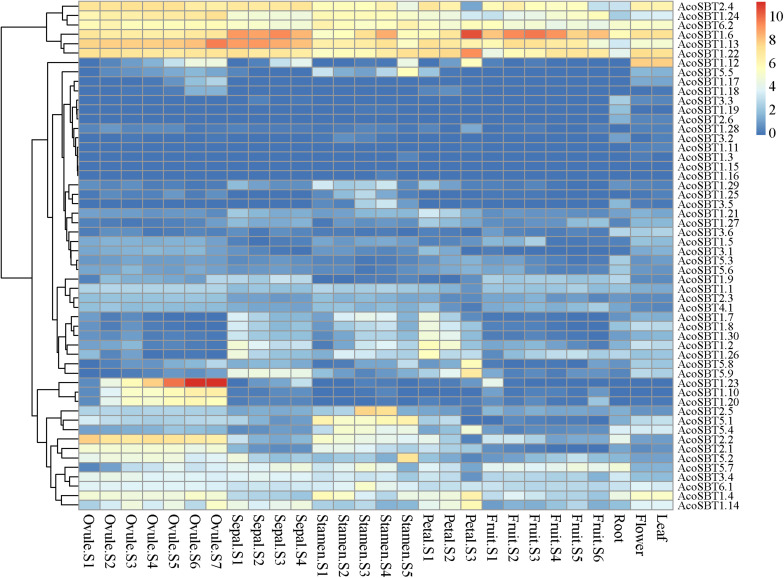
Tissue-specific expression profiles of AcoSBTs in pineapple. Heat-map of tissue-specific expression profiles of AcoSBTs in pineapple. Samples are mentioned at the top of each lane: ovule S1–S7, sepal S1–S4, stamen S1–S5, petal S1–S3, root, leaf, flower, fruit S1–S7. “S” is the abbreviation of the word “stage.”

To explore the possible role of *AcoSBT* genes in pineapple growth and development, we analyzed the expression profiles of all *SBT* genes in different tissues and developmental stages using publicly available transcriptome databases. The 54 *AcoSBT*s exhibit diverse organ-specific expression patterns, which may help to illustrate the functional divergence of SBT gene family genes in pineapple growth and development (Supplementary Table 5). As shown in Figures 3, 6 Group I genes, *AcoSBT1.23*, *AcoSBT1.10*, and *AcoSBT1.20*, were specifically expressed in 5 developmental stages of pineapple female sexual organ ovule. In contrast, 2 Group V genes, *AcoSBT5.1* and *AcoSBT5.4*, had analogical transcription profiles that were specifically expressed in pineapple stamen. Those results implicate that Group I and Group V genes might play essential roles in the female and male gametophyte development, respectively. In Group III, almost all genes except *AcoSBT3.4* were poorly expressed in all tested samples, illustrating that the genes in this group might not be involved in the developmental processes in pineapple. Meanwhile, 6 genes (*AcoSBT2.4*, *AcoSBT1.24*, *AcoSBT6.2*, *AcoSBT1.6*, *AcoSBT1.13*, and *AcoSBT1.22*) were ubiquitously expressed in all tested tissues (7 ovule developmental tissues, 4 sepal developmental tissues, 6 stamen developmental tissues, 3 petal developmental tissues, 6 fruit developmental tissues, as well as root, flower and leaf samples), suggesting that these 6 genes might exert necessary functions for both vegetative and reproductive growth in pineapple.

We also verified the reliability of transcriptome data using qRT-PCR technology. In this analysis, the mixed stage samples of 5 tissues (leaf, sepal, petal, stamen, and ovule) were used to investigate the transcription of 12 abundantly or specifically expressed genes, namely, *AcoSBT1.12*, *AcoSBT1.13*, *AcoSBT1.22*, *AcoSBT1.23*, *AcoSBT1.24*, *AcoSBT1.4*, *AcoSBT1.6*, *AcoSBT2.2*, *AcoSBT2.4*, *AcoSBT2.5*, *AcoSBT5.2*, and *AcoSBT6.2*. The results showed that the expression patterns of qRT-PCR were generally consistent with that given by RNA-seq analysis (Supplementary Figure 3).

### Subcellular Localization of AcoSBT1.12 Proteins

The *Cis*-acting elements profile showed that *AcoSBT1.12* harbored various *Cis*-acting elements in its promoter region, and the expression pattern revealed that *AcoSBT1.12* specifically expresses in flower and leaf, suggesting that *AcoSBT1.12* might play an essential role in the development of above-ground organs in pineapple. As previously reported, *SBTI* genes play essential roles in plant growth and development (Schaller et al., 2012). According to the phylogenetic tree constructed in this study, *AcoSBT1.12* was clustered into the subclade with widely reported *SBTI* genes.

**FIGURE 7:**
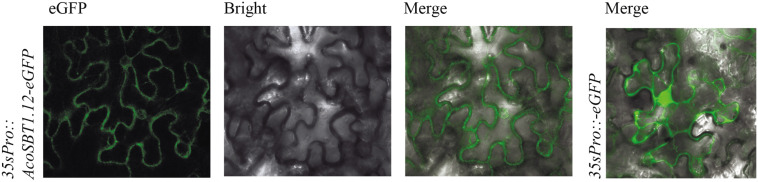
Subcellular location of AcoSBT1.12. The subcellular location of AcoSBT1.12 and blank vector were detected on the surface cells of *Nicotiana Benthamiana’*s leave.

To further investigate the subcellular localization of AcoSBT1.12 in plant cells, We fused the coding sequence of *AcoSBT1.12* to the area between the *CaMV 35S* promoter and the GFP encoding region. The transient expression leaves in tobacco (*Nicotiana benthamiana*) showed that the AcoSBT1.12-GFP protein was mainly distributed in the plasma membrane, whereas the control protein produced by the empty vector (35S:GFP) was equally distributed in the plasma membrane and the nucleus (Figure 7).

### AcoSBT1.12 Delays Floral Transition in Arabidopsis

**FIGURE 8:**
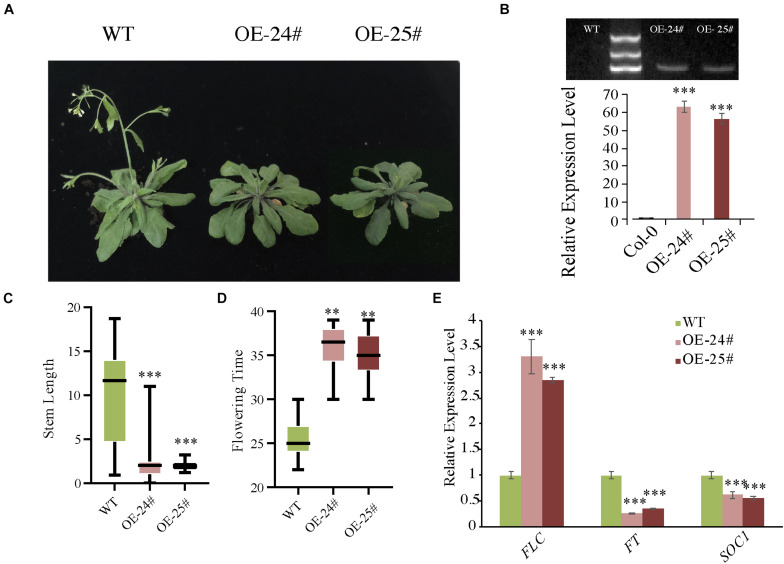
Overexpression of *AcoSBT1.12* in Arabidopsis delayed floral transition with the expression level of *FLC* increased while *FT* and *SOC1* decreased. **(A)** Characterization of the flowering phenotype of *AcoSBT1.12* OE lines. **(B)** The gel electrophoresis results and qPT-PCR results confirmed the reliability of OE lines. **(C)** Stem length on the 40th day, the error bars indicate + SD. **(D)** Flowering time of WT and OE lines, the error bars indicate ± SD. **(E)** Relative expression level of flowering regulator genes. Asterisks indicate significant differences for the indicated comparisons based on Student’s *t*-test (^∗∗∗^*p* < 0.001; ^∗∗^*p* < 0.01).

To further confirm the hypothesis that AcoSBT1.12 is involved in plant growth, we overexpressed AcoSBT1.12 in Arabidopsis driving by 35S promoter (Supplementary Table 6). Two *AcoSBT1.12* T3 transgenic lines (OE-24# and OE-25#) with positive PCR bands and high *AcoSBT1.12* transcription abundance were selected for phenotyping (Figure 8).

Under LD (Long-day) conditions, transgenic lines showed delayed floral transition with better-developed rosette leaves than WT (Figures 8A,C). Normally, WT plants bolt in about 25 days, while the overexpressing lines are delayed by about 10 days (Figure 8D). Meanwhile, the expression levels of three critical floral transition regulating genes were changed significantly. The MADS-box gene *FLC* (*FLOWERING LOCUS C*), the inhibitor of flowering event, was significantly up-regualated in all three OE lines (Figure 8E). While *FT* (*FLOWERING LOCUS T*) and *SOC1* (*SUPPRESSOR OF OVEREXPRESSION OF CONSTANS1*), function in flowering induction in LD, were down-regulated in the three OE lines (Figure 8E). However, under SD (Short day) conditions, the transgenic lines didn’t show any difference from WT plants. Collectively, these results indicate that *AcoSBT1.12* might participate in floral transition under LD conditions.

**FIGURE 9:**
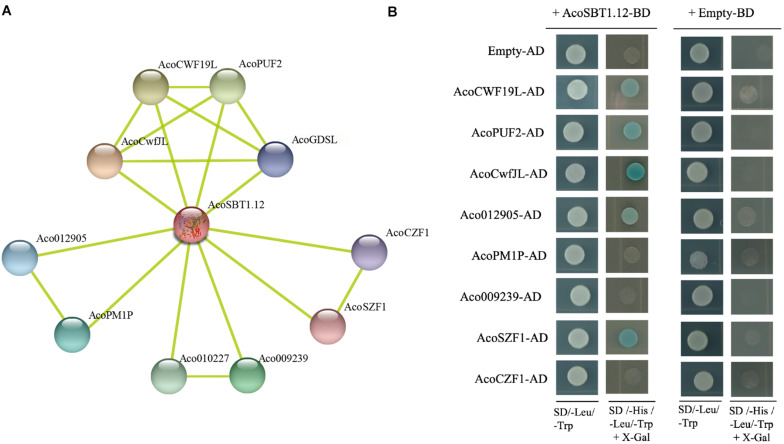
*AcoSBT1.12* influences flowering time. **(A)** The network prediction of AcoSBT1.12 and its interaction protein based on STRING database. **(B)** Protein interactions between AcoSBT1.12 and predicted protein by yeast two-hybrid assay.

Since the *SBT* family genes encode proteases, identifying the potential substrates of AcoSBTs will help to understand their function further. Using STRING database, 10 pineapple proteins that might interact with AcoSBT1.12 were predicted. Subsequently, the coding sequences of eight of these genes (*AcoCWF19L*, *AcoPUF2*, *AcoCwfJL*, *Aco012905*, *AcoPM1P*, *Aco009239*, *AcoSZF1*, and *AcoCZF1*) were fused into the pGADT7 as AD constructs, while the CDS of *AcoSBT1.12* was cloned into the pGBDT7 as BD construct. Yeast two-hybrid assay showed that AcoSBT1.12 could interact with AcoCWF19L, AcoPUF2, AcoCwfJL, Aco012905, and AcoSZF1 (Figure 9 and Supplementary Table 6). Although litter is known about these proteins, these conformed protein-protein interactions will provide reference information for studying the regulatory functions of *AcoSBT* genes in pineapple.

## Discussion

It is extensively reported that SBT proteins exist in all three domains of life and are relatively conserved across different plant species. Beginning from the first description of 15 *SBT* genes in tomato (*Lycopersicon esculentum*), the gene members from this family were further identified and well-characterized in many dicotyledons, including Arabidopsis (56 gene members), poplar (*Populus trichocarpa*, 90), potato (*Solanum tuberosum*, 82), and melon (*Cucumis melo* L.) (Yamagata et al., 1994; Meichtry et al., 1999; Rautengarten et al., 2005; Tian et al., 2005; Schaller et al., 2012; Norero et al., 2016). In this study, 54 *AcoSBTs* were identified via the HMM-based method in the pineapple genome. Compared with polar and potato, the pineapple SBT family is relatively small, suggesting that some *SBT* members may have undergone an evolutionary selection and get lost in pineapple during evolution. To reveal the phylogenetic relationship of pineapple *SBT* genes, a phylogenetic tree was constructed, and the pineapple SBT members were classified into 6 groups according to their phylogenetic relationship (Figure 1A).

In the subsequent studies on gene structure and motif composition, we further proved the relative conservation among the members of the same subfamily (Figure 2). The investigation of gene duplication events illustrated the potential expansion mechanism of *AcoSBT*s and showed that the duplication gene pairs tend to be from the same subfamily (Figures 3, 4). Subsequent *Cis*-acting element analysis showed that AcoSBTs probably function in light-responsiveness events. The expression pattern analysis showed that the expression patterns of *AcoSBT* genes showed an organ-specific tendency (Figures 5, 6), which is consistent with the previous findings in Arabidopsis and rice. These results indicated that the *AcoSBT* genes might be regulated by light and might participate in many aspects of photomorphogenesis in pineapple (Jonesb et al., 2004). It has been previously reported that several Group I members are involved in many aspects of plant growth and development, including embryo development, stomatal density regulation, and reproductive development. For example, *SBT1.12*, also named as *SDD1* represent for *Stomatal Density and Distribution 1*, highly expressed in stomata initials but undetectable in mature guard cells, functions in stomata development suppression via *TMM* (*Too Many Mouths*)—dependent pathway in both Arabidopsis and *Solanum lycopersicum* (Groll et al., 2002; Morales et al., 2018). In Arabidopsis, *SBT1.4* (*SASP*), expressed in all above-ground organs, down-regulates silique production and branched inflorescences during reproductive development (Wang et al., 2018). In legumes, including *Medicago truncatula* and *Pisum sativum*, SBT1.1 proteins locate in the endosperm, controlling seed size variation through regulating embryo cell division during reproductive development (D’Erfurth et al., 2012). The apparent relevance of *SBT I* subfamily with plant development, together with the significant expansion and uneven distribution of *SBT I* members in pineapple genome indicates the unique role of this gene subfamily in pineapple development.

Among all *AcoSBT I* members, the transcription level of *AcoSBT1.12* was relatively high in leave and flower. Interestingly, we also found AcoSBT1.12 shared conserved domains with SDD and SASP in many species, both of which are involved in above-ground organ development (Groll et al., 2002; Morales et al., 2018). These results suggested that *AcoSBT1.12* genes may have functions in pineapple growth and development. Due to the limitations of pineapple transformation technology, we constructed *AcoSBT1.12* OE lines in Arabidopsis. Our results showed that overexpression of pineapple *AcoSBT1.12* delayed the flowering time of Arabidopsis under LD conditions. Moreover, The core flowering regulating genes, *FT*, *FLC*, and *SOC1*, were up-regulated in the transgenic Arabidopsis OE lines (Figure 8). In angiosperms, the flowering transition located at the converter node from vegetative growth to reproductive growth is the most vital phase transition in plant development (Domagalska et al., 2010). Plant flowering is regulated by six major flowering genetic pathways to shape maximum reproductive success, including photoperiod, thermosensory, age, autonomous, vernalization, and GA pathways (Coupland, 1995; Koornneef et al., 1998; Fornara et al., 2010). The MADS-box gene *FLC* (*FLOWERING LOCUS C*), the first reported gene regulating flowering transition, locating in the central position of the flowering network, acts as a canary in the coal mine (Sheldon et al., 2000; Mentzer et al., 2010). And *FT* (*FLOWERING LOCUS T*), another widely studied florigen in the flowering regulatory network, is also acknowledged as the basis of numerous signaling pathway directly affect floral transition (Liu et al., 2007; Eckardt, 2010). Ectopic expression of *AcoSBT1.12* affected the expression abundance of *FT* and *FLC* in Arabidopsis, which confirmed that *SBT1*.12 is involved in floral transition controlling in plants. Base on bioinformatics analysis, we conducted a yeast two-hybrid experiment and confirmed the interaction relationships between AcoSBT1.12 and PLANT-UNIQUE RAB5 EFFECTOR 2 (AcoPUF2). Due to the lack of molecular biological and genetic studies done in pineapple, further research needs to be done to elusive the molecular mechanism underlying AcoSBT1.12-mediated flowering transition. However, the valuable information in this study will shed light on the understanding of SBT-involved development in pineapple.

## Conclusion

In this study, 54 *AcoSBT*s were firstly characterized base on genome-wide identification of the SBT gene family in pineapple. The results of the evolution scenario analysis of *AcoSBT*s suggested that *AcoSBT*s are highly conserved compared with their homologs from other monocotyledon plants. In addition, the expression profiling accompany with Cis-elements analysis showed that *AcoSBT* genes have essential roles in controlling plant growth and development. Overexpression of *AcoSBT1.12* in Arabidopsis delayed flowering time and altered the expression level of *FLC*, *FT*, and *SOC1*. In conclusion, these results provide valuable information for further studying the roles of *AcoSBT*s in plant development and growth.

## Data Availability Statement

The datasets presented in this study can be found in online repositories. The names of the repository/repositories and accession number(s) can be found below: https://www.ebi.ac.uk/ena, PRJEB38680.

## Author Contributions

XJ performed vector construction and phenotype analysis. XJ and YL performed RNA-seq and transformation. YZ, ZH, and YF calculated all the data. ZH and HC performed qRT-PCR analysis. XJ and YQ wrote the manuscript. YC, YQ, and HC revised the manuscript. All authors have read and agreed to the published version of the manuscript.

## Conflict of Interest

The authors declare that the research was conducted in the absence of any commercial or financial relationships that could be construed as a potential conflict of interest.

## Publisher’s Note

All claims expressed in this article are solely those of the authors and do not necessarily represent those of their affiliated organizations, or those of the publisher, the editors and the reviewers. Any product that may be evaluated in this article, or claim that may be made by its manufacturer, is not guaranteed or endorsed by the publisher.

## Footnotes

1
http://pfam.xfam.org/

2
https://phytozome-next.jgi.doe.gov

3
https://www.ncbi.nlm.nih.gov/cdd

4
http://web.expasy.org

5
http://www.cbs.dtu.dk.services/TargetP/

6
http://www.cbs.dtu.dk.services/SignalP/

7
http://www.arabidopsis.org

8
https://itol.embl.de

9
http://meme-suite.org/tools/meme

10
http://bioinformatics.psb.ugent.be/webtools/plantcare/html

11
http://www.ncbi.nlm.nih.gov/Traces/study/?acc=SRP067011

## Supplementary Material

Supplementary Figure 1 The detailed logo of each letter represents the level of conservation of amino acid of each Motifs.

Supplementary Figure 2 Distribution of motifs in promoter region of each gene.

Supplementary Figure 3 Validation of *AcoSBT* genes expression profile by qRT-PCR at five different tissues.

Supplementary Table 1 Detailed information of pineapple SBTs, including sequenced ID, chromosome locations, isoelectric point (pI), molecular weight (KD), protein length and function domains.

Supplementary Table 2 The multilevel consensus sequence and description of 10 motifs.

Supplementary Table 3 The original data of Ka and Ks value.

Supplementary Table 4 The distribution of Cis-acting elements.

Supplementary Table 5 The original data of FPKM value.

Supplementary Table 6 Information about *AcoSBT1.12* and genes encoding interacting proteins.
