# Supplementary figures and images for: Genome-Wide Investigation of SBT Family Genes in Pineapple and Functional Analysis of AcoSBT1.12 in Floral Transition
Source: Front Genet. 2021 Sep 7;12:730821. doi: 10.3389/fgene.2021.730821 (PMC8452990; doi:10.3389/fgene.2021.730821)

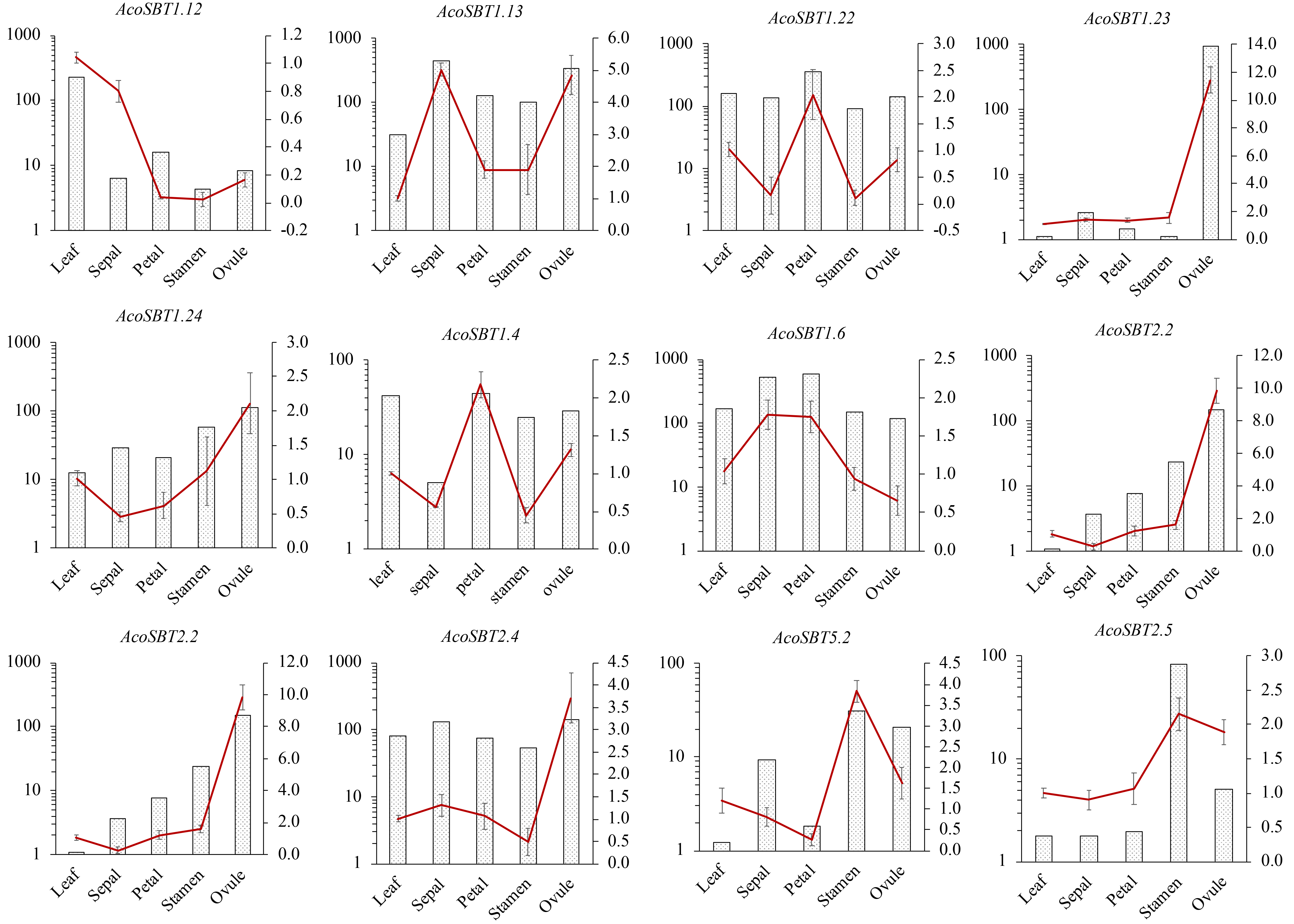

Supplement: Supplementary Figure 1 — The detailed logo of each letter represents the level of conservation of amino acid of each Motifs. [file Image_1.TIF]

Motif 1

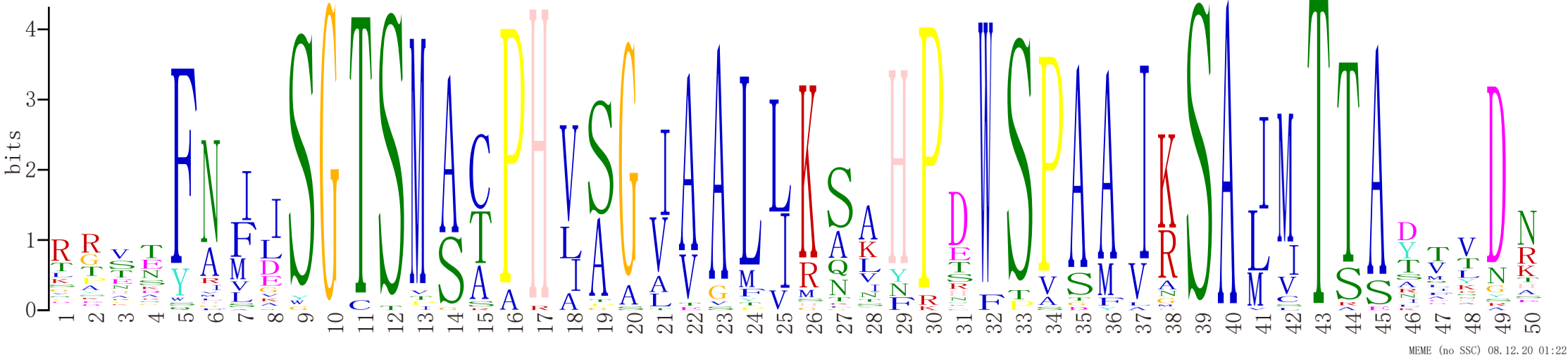

Motif 2

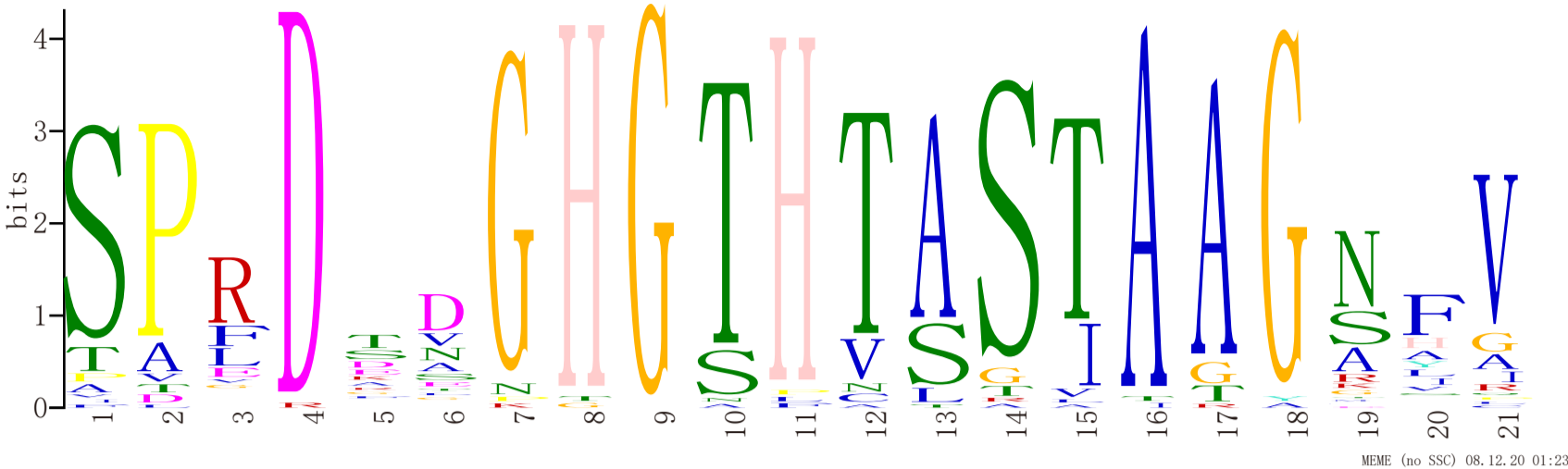

Motif 3

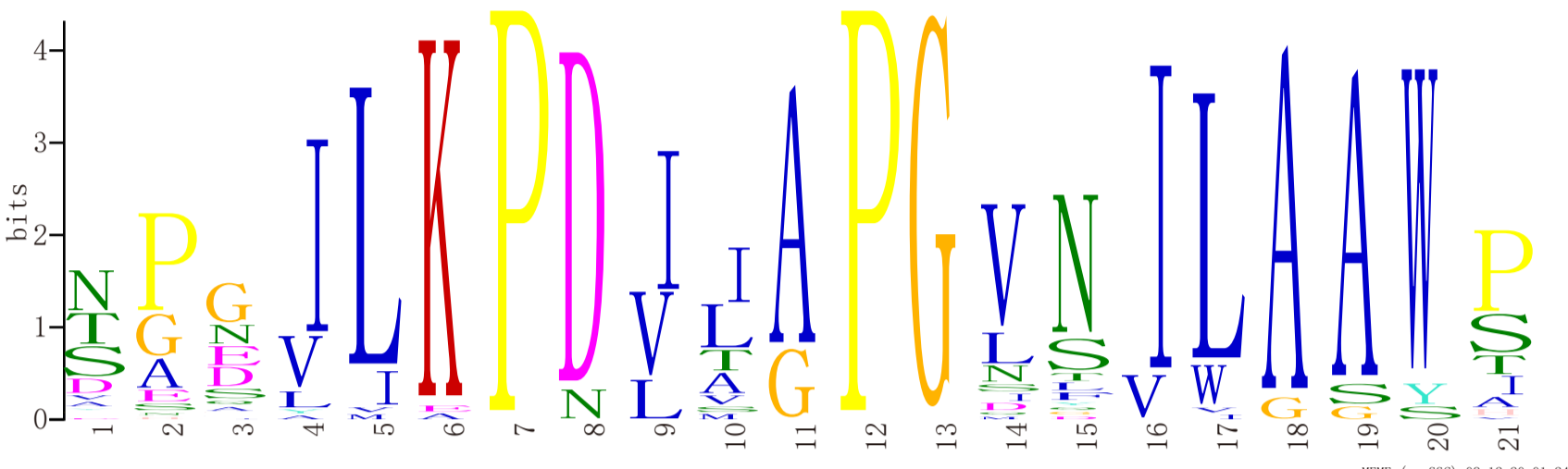

Motif 4

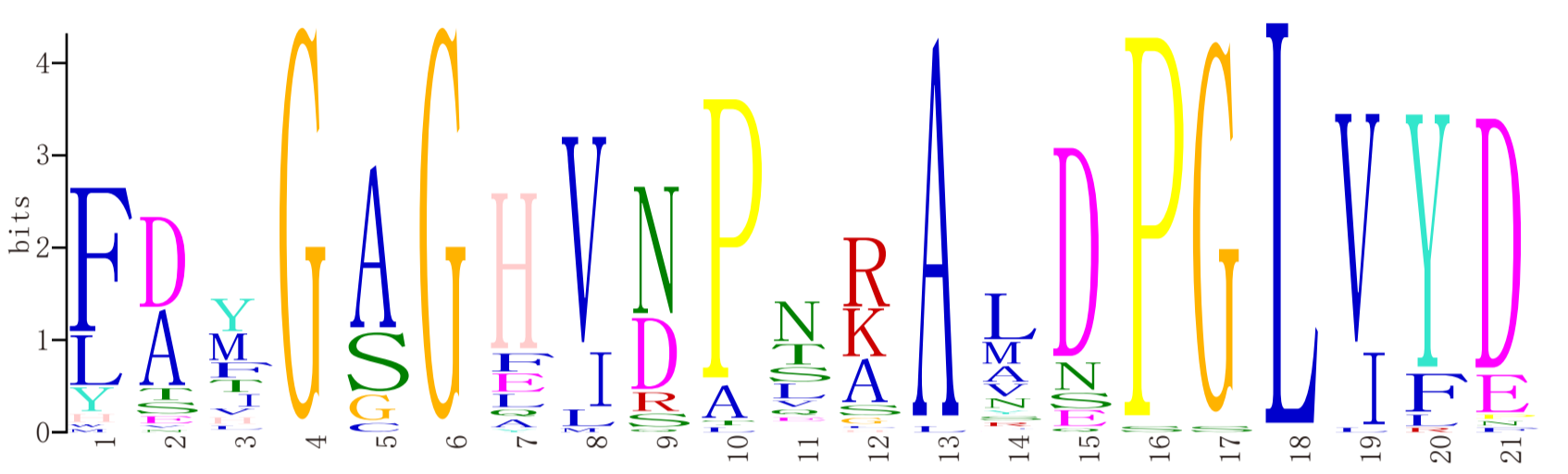

Motif 5

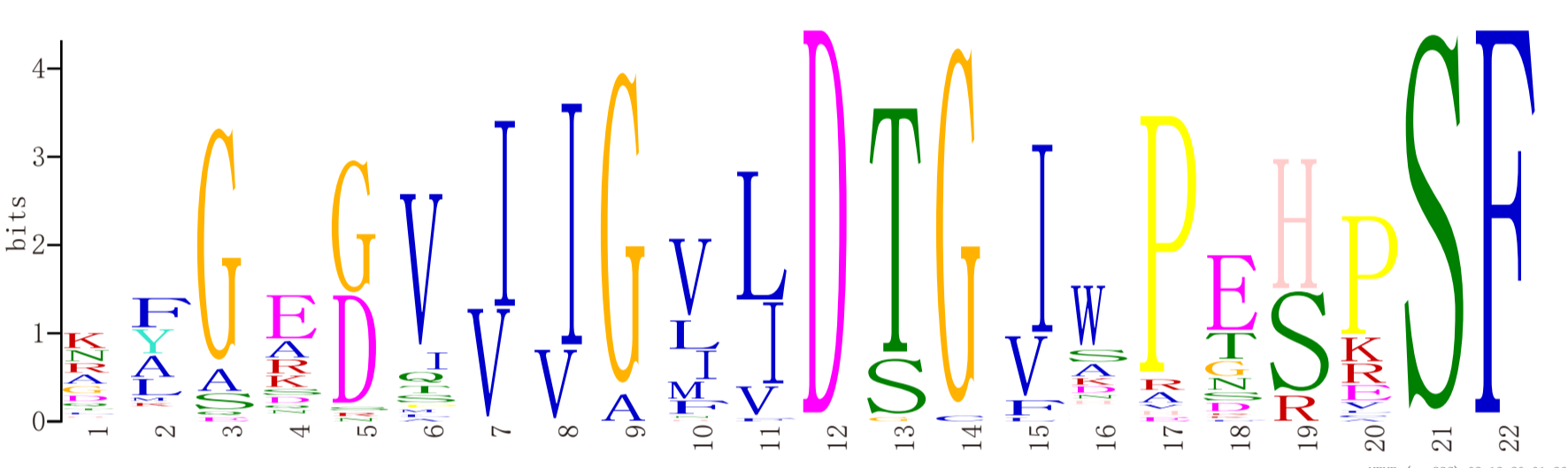

Motif 6

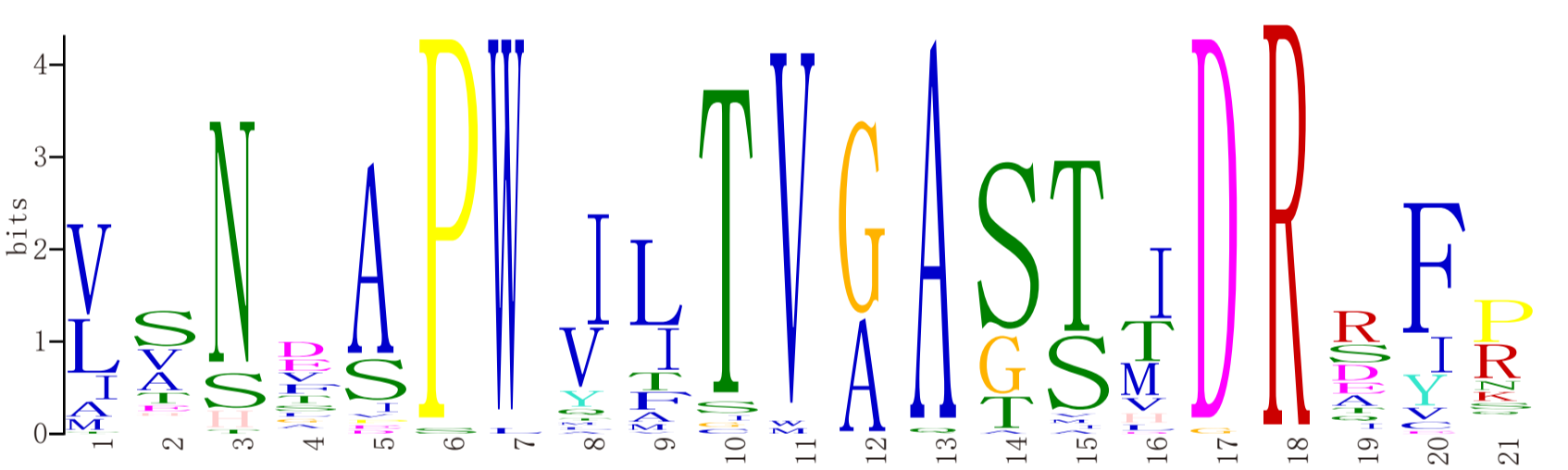

Motif 7

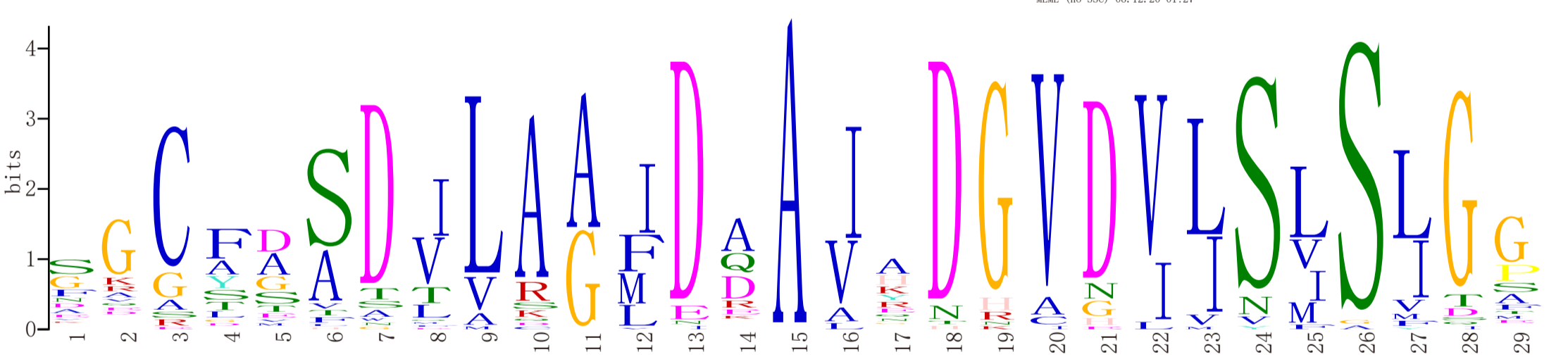

Motif 8

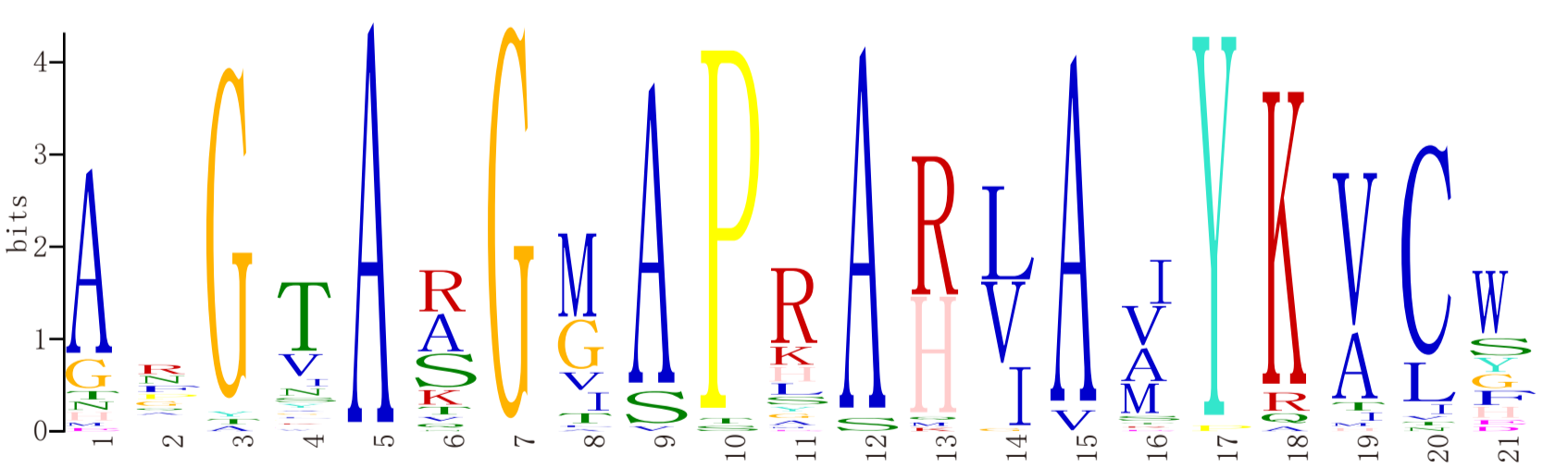

Motif 9

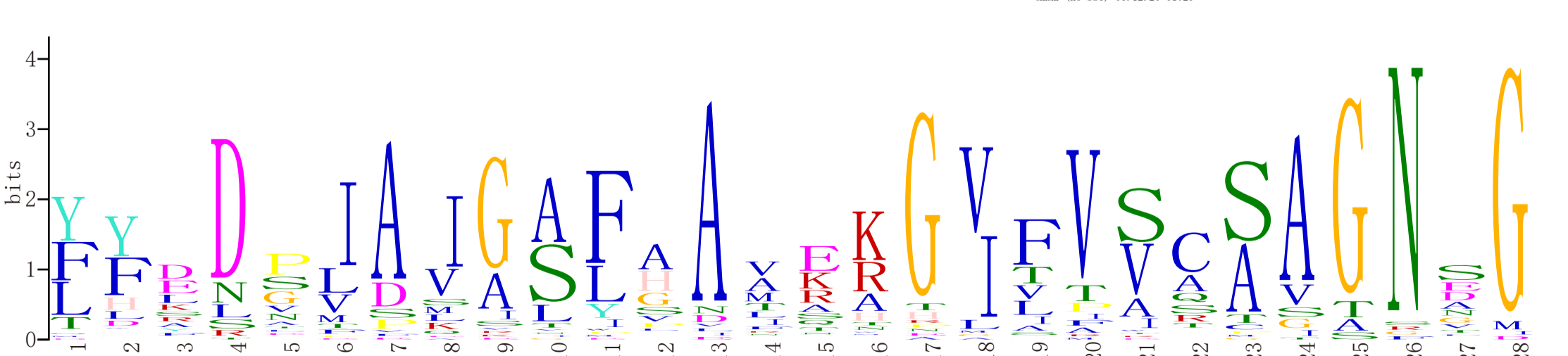

Motif 10

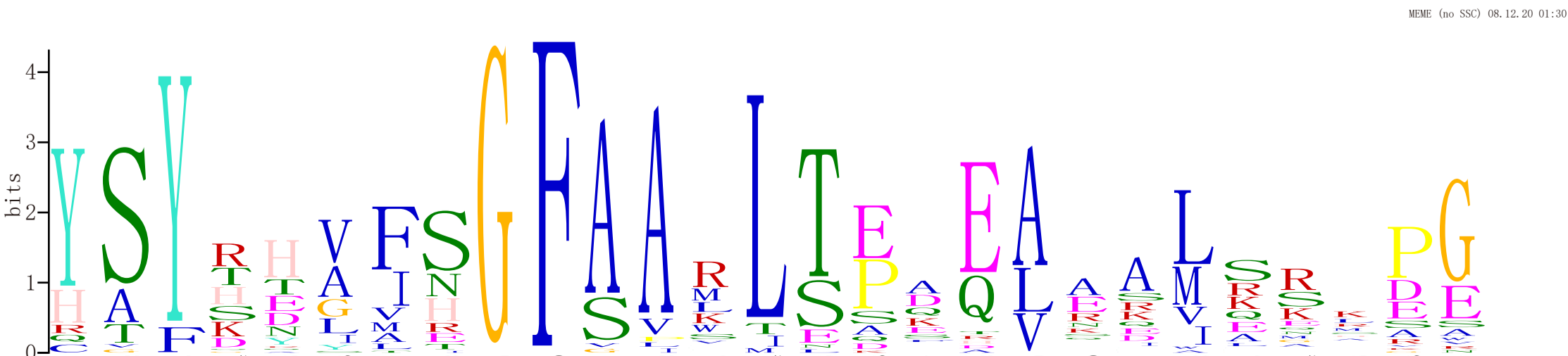

Supplement: Supplementary Figure 2 — Distribution of motifs in promoter region of each gene. [file Data_Sheet_1.PDF]

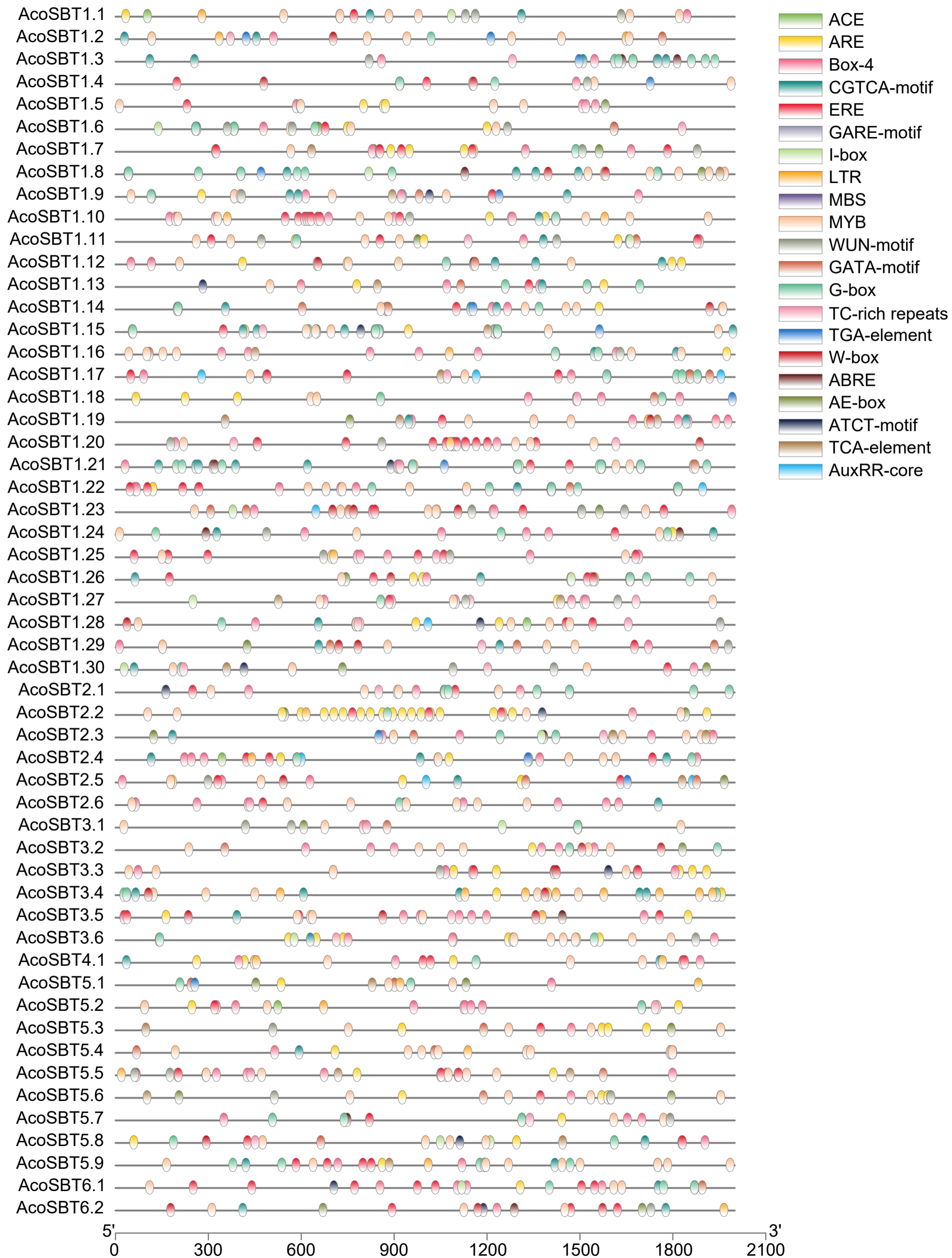

Supplement: Supplementary Figure 3 — Validation of AcoSBT genes expression profile by qRT-PCR at five different tissues. [file Data_Sheet_2.PDF]
